# Development and Validation of an Internationally-Standardized, High-Resolution Capillary Gel-Based Electrophoresis PCR-Ribotyping Protocol for *Clostridium difficile*

**DOI:** 10.1371/journal.pone.0118150

**Published:** 2015-02-13

**Authors:** Warren N. Fawley, C. W. Knetsch, Duncan R. MacCannell, Celine Harmanus, Tim Du, Michael R. Mulvey, Ashley Paulick, Lydia Anderson, E. J. Kuijper, Mark H. Wilcox

**Affiliations:** 1 Department of Microbiology, Leeds Teaching Hospitals NHS Trust, Leeds, United Kingdom; 2 Centers for Disease Control and Prevention (CDC), Atlanta, United States of America; 3 Department of Medical Microbiology, Centre of Infectious Diseases, Leiden University Medical Centre, Leiden, Netherlands; 4 Public Health Agency of Canada (PHAC), Winnipeg, Canada; 5 Leeds Institute of Molecular Medicine, University of Leeds, Leeds, United Kingdom; University Medical Center Groningen, NETHERLANDS

## Abstract

PCR-ribotyping has been adopted in many laboratories as the method of choice for *C. difficile* typing and surveillance. However, issues with the conventional agarose gel-based technique, including inter-laboratory variation and interpretation of banding patterns have impeded progress. The method has recently been adapted to incorporate high-resolution capillary gel-based electrophoresis (CE-ribotyping), so improving discrimination, accuracy and reproducibility. However, reports to date have all represented single-centre studies and inter-laboratory variability has not been formally measured or assessed. Here, we achieved in a multi-centre setting a high level of reproducibility, accuracy and portability associated with a consensus CE-ribotyping protocol. Local databases were built at four participating laboratories using a distributed set of 70 known PCR-ribotypes. A panel of 50 isolates and 60 electronic profiles (blinded and randomized) were distributed to each testing centre for PCR-ribotype identification based on local databases generated using the standard set of 70 PCR-ribotypes, and the performance of the consensus protocol assessed. A maximum standard deviation of only ±3.8bp was recorded in individual fragment sizes, and PCR-ribotypes from 98.2% of anonymised strains were successfully discriminated across four ribotyping centres spanning Europe and North America (98.8% after analysing discrepancies). Consensus CE-ribotyping increases comparability of typing data between centres and thereby facilitates the rapid and accurate transfer of standardized typing data to support future national and international *C. difficile* surveillance programs.

## Introduction

*Clostridium difficile* infection (CDI) is a major nosocomial disease, placing a considerable burden on healthcare resources. A recent European report estimated the associated per-case cost of CDI at £4577–£8843 [1]. *C*. *difficile* has commonly been associated with hospital outbreaks, and most notably the epidemic spread of NAP1/BI/PCR ribotype 027 [2, 3]. Studies of *C*. *difficile* epidemiology from diverse geographical locations have described different locally or nationally prevalent strains [4, 5, 6], some of which were associated with increased severity and/or reduced antimicrobial susceptibility [2, 5]. DNA typing systems have been crucial in tracking the emergence and spread of *C*. *difficile* strains. Notably, individual surveillance programs have seen significant changes in the prevalence and epidemiology of NAP1/BI/PCR ribotype 027 [7, 8]. This highlights the important role that DNA typing systems play in epidemiological surveillance and in targeting informing infection control interventions.

Most epidemiological studies of *C*. *difficile* are local or national in scope, and as such have used different typing techniques. Pulsed-field gel electrophoresis (PFGE) [9], restriction enzyme analysis (REA) [8], PCR-Ribotyping [7], toxinotyping [10], arbitrary-primed PCR (AP-PCR) [11], random amplification of polymorphic DNA (RAPD) [12] and REP-PCR [13] have all been used routinely for *C*. *difficile* typing; thus, it is not uncommon for isolates to be referred to by multiple typing designations (eg: NAP1/BI/027; PFGE/REA/PCR-ribotyping). This can be problematic when trying to compare studies or conduct large scale epidemiological analyses. A multi-centre, international study assessed seven typing techniques, and drew attention to the lack of a consensus technique with proven inter-laboratory reproducibility [14]. PCR ribotyping has gained acceptance in Europe as the typing method of choice, with a common nomenclature adopted across most countries (although some local systems have emerged) and different primers can be employed [15, 16, 17]. Agarose gel-based methods offer relatively high sensitivity, specificity and reproducibility relative to other PCR-based techniques, but inter-laboratory variation in banding patterns and interpretation mean that data are difficult to compare between typing centres [14].

The advent of high-resolution, capillary gel electrophoresis-based fragment analysis addressed many of these issues. Reduced PCR cycling and electrophoresis times combined with the ability to increase batch sizes (using multiple fluorescent detection dyes) represents a relatively rapid, high-throughput and low cost means of epidemiological *C*. *difficile* investigation [18, 19, 20]. However, CE-ribotyping has yet to gain widespread acceptance, and is hampered by a lack of multi-centre evaluations demonstrating its reliability and the portability of data. Indra *et al*. proposed and established a web-based repository of electronic *C*. *difficile* PCR ribotyping data (WebRibo) [18]. Unfortunately, the lack of protocol standardization and consequent limited capacity to accurately categorize submitted patterns using internationally recognised PCR-ribotype nomenclature has to date diminished the effectiveness of this approach [21]. We describe here the first multi-centre development, validation and evaluation of a standardised protocol for high-resolution PCR-ribotyping using capillary gel electrophoresis.

## Materials and Methods

### Isolates

A panel of well characterised *C*. *difficile* isolates representing 70 distinct PCR-ribotypes was used in this study [22]. The panel comprised PCR-ribotypes known to be associated with human CDI in Europe. This collection was assembled using type strains previously shared between two established PCR-ribotyping laboratories (*Clostridium difficile* Network for England and Northern Ireland (CDRN), and National Reference Laboratory for *Clostridium difficile* at University Medical Centre, Leiden). All PCR-ribotypes were originally assigned in association with the Anaerobic Reference Laboratory at Cardiff (ARL) using agarose gel-based PCR-ribotyping technique (Table 1). Data on isolates in the panel have been made available on-line in a National Center for Biotechnology Information BioProject database (NCBI) (http://www.ncbi.nlm.nih.gov/bioproject/248340). In addition, a subset (European Centre for Disease Prevention and Control (ECDC)-Brazier collection) is available to all reference laboratories in Europe who participate in the European *C*. *difficile* infection study network (ECDIS-NET) [23].

**Table 1:** PCR-ribotypes represented in the study and identification performance of the testing laboratories at subsequent validation stages of the CE-ribotyping protocol.

PCR-ribotypes involved in the study	VALIDATION STAGE 1 Training set PCR-ribotypes represented	VALIDATION STAGE 2 Challenge set (isolates)		VALIDATION STAGE 3 Challenge set (electronic data)	
		PCR-ribotype represented	Identification score^1^	PCR-ribotype represented	Identification score^1^
001	001	001 (x2)^2^	4/4; 4/4	001	4/4
002	002	002	4/4	002	4/4
003	003	003	4/4	003	4/4
004	004	004	4/4	004	4/4
005	005	005	4/4	005	4/4
006	006	*not included*	-	006	4/4
007	007	007	4/4	007	4/4
009	009	*not included*	-	009	4/4
010	010	010	4/4	010	3/4
011	011	011	4/4	011	4/4
012	012	012	4/4	012	3/4
014	*not included*	014	4/4 Unrecognised profile*	*not included*	-
015	015	015	4/4	015	4/4
016	016	016	4/4	016	4/4
017	017	017	4/4	017	4/4
018	018	018	4/4	*not included*	-
019	019	019	4/4	019	4/4
020	020	*not included*	-	020	4/4
023	023	023	4/4	023	4/4
025	025	025	4/4	025	4/4
026	026	026	4/4	026	4/4
027	027	027	4/4	027	4/4
029	029	029	3/4	029	4/4
031	031	*not included*	-	031	4/4
033	033	033	4/4	033	4/4
035	035	035	3/4	035	4/4
037	037	037	4/4	037	4/4
040	040	*not included*	-	040	4/4
042	042	042	4/4	042	4/4
043	043	043	4/4	043	4/4
045	045	*not included*	-	045	4/4
046	046	046	4/4	046	4/4
047	047	*not included*	-	047	4/4
050	050	*not included*	-	050	4/4
051	051	051	4/4	051	4/4
052	052	*not included*	-	*not included*	-
053	053	053	4/4	053	4/4
054	054	054	4/4	054	4/4
055	055	055	4/4	055	4/4
056	056	*not included*	-	056	4/4
057	057	*not included*	-	057	4/4
058	058	058	3/4	058	4/4
060	060	060	4/4	060	4/4
062	062	062	4/4	062	4/4
063	063	063	4/4	063	3/4
064	064	*not included*	-	064	4/4
066	066	066	4/4	066	4/4
067	067	067	4/4	067	4/4
068	068	068	4/4	*not included*	-
070	070	070	4/4	070	4/4
072	072	*not included*	-	*not included*	-
075	075	075	4/4	075	4/4
076	076	*not included*	-	076	4/4
077	077	*not included*	-	*not included*	-
078	078	078	4/4	078	4/4
079	079	*not included*	-	079	4/4
081	081	081	4/4	081	4/4
083	083	083	4/4	*not included*	-
084	084	*not included*	-	084	4/4
085	085	*not included*	-	085	4/4
087	087	087	4/4	087	4/4
095	095	*not included*	-	*not included*	-
106	106	106	4/4	106	4/4
118	118	118	3/4	*not included*	-
122	122	122	4/4	122	4/4
126	126	*not included*	-	126	4/4
131	131	131	4/4	*not included*	-
153	153	*not included*	-	*not included*	-
169	169	*not included*	-	169	4/4
174	174	174	4/4	174	4/4
198	198	036	3/4 Unrecognised profile*	198	4/4

^1^ Number of laboratories that identified the correct PCR-ribotype (or correctly stated that the profile generated was not a recognisable PCR-ribotype present in the training set)

^2^ two individual PCR-ribotype 001 isolates were present in the challenge (isolates) set

*Correctly identified that the profile was not present in the training set

### Development of consensus capillary gel electrophoresis-based PCR-ribotyping

Protocols from each of the four participating laboratories were initially compared. Reagents, PCR conditions, sequencer model, polymer, array length and separation parameters were recorded. A consensus protocol was developed by comparing and contrasting the four internal protocols and was tested internally against the panel of reference strains to evaluate performance. Following refinements, a consensus protocol was distributed to the four participating national laboratories for feedback, testing and validation. Individual internal protocols and the final consensus protocol are summarised in Table 2.

**Table 2:** Comparison of internal protocols from the four participating laboratories and the proposed consensus protocol.

Centre	Primer set	Polymerase	Thermal cycling conditions	Electrophoresis	Size marker
	STUBBS^1^		5m @95°C		
		HotStarTaq Plus Mastermix	[60s@92°C, 60s@55°C, 90s@72°C]x26	3130 instrument	GeneScan 600/1200 LIZ (LIZ)
**A**		(Qiagen)	60s@95°C	36cm array length	(Applied Biosystems)
			45s@55°C	POP 7 polymer	[20–600/1200bp]
			5m@72°C		
	BIDET^2^	HotStarTaq	15m @ 95°C	3130 instrument	Geneflo 625 (ROX)
**B**		(Qiagen)	[60s@95°C, 60s@57°C, 60s@72°C)]x35	36cm array length	Chimerx
			5m @ 72°C	POP 7 polymer	[50–625bp]
	BIDET^2^	AccuPrime High Fidelity	5m @95°C	3130xl instrument	GeneScan 1200 LIZ (LIZ)
**C**		(Invitrogen)	[60s@95°C, 60s@57°C, 60s@72°C]x35	36cm array length	(Applied Biosystems)
			30m@72°C	POP 7 polymer	[20–1200bp]
	BIDET^2^	HotStarTaq Plus Mastermix	5m @95°C	3100instrument	MapMarker 1000 (ROX)
**D**		(Qiagen)	[60s@95°C, 60s@57°C, 60s@72°C]x17	36cm array length	(BioVentures)
			30m@72°C	POP 4 polymer	[50–1000bp]
**PROPOSED**	BIDET^2^	HotStarTaqMastermix	5m @95°C	3130xl instrument	GeneScan 1200 LIZ (LIZ)
**CONSENSUS**		(Qiagen)	[60s@95°C, 60s@57°C, 60s@72°C]x26	36cm array length	(Applied Biosystems)
**PROTOCOL**			30m@72°C	POP 7 polymer	[20–1200bp]

^1^Stubbs *et al*., 1999 (16)

^2^Bidet *et al*., 1999 (17)

### DNA extraction

DNA was extracted from *C*. *difficile* cultures using Chelex-100 resin. Briefly, several colonies of test organism, taken from fresh 24 h cultures on a non-selective solid medium, were fully resuspended in 100μL 5% w/v Chelex-100 resin (Bio-Rad, Hertfordshire, UK) in molecular grade H_2_O. The bacterial suspension was heated at 100°C for 10 min and the resultant lysate was centrifuged for 2 min at 13,000 rpm. The supernatant was collected and DNA concentration was adjusted to 100ng/μL and used immediately for PCR.

### Amplification of 16S-23S intergenic spacer region

PCR primers 5’-GTGCGGCTGGATCACCTCCT-3’ (16S) and 5’-CCCTGCACCCTT-AATAACTTGACC-3’) (23S) were used for amplification [17]. The 16S primer was labelled at the 5’ end with either 6-carboxyfluorescein (6-FAM) or 5-tetrachlorofluorescein (TET). Individual PCR reactions comprised 2 μL prepared bacterial DNA, 0.2 uM each primer and 12.5 uL HotStarTaq mastermix (Qiagen, Manchester, UK). Reactions were made up to 25 μL with molecular grade water. Conventional thermal cycling was as follows: initial polymerase activation at 95°C for 15 min, followed by 24cycles at 95°C for 1 min, 57°C for 1 min and 72°C for 1 min, with a final completion stage at 72°C for 30 min.

### Capillary gel electrophoresis

PCR products were analysed on either an ABI 3100 or 3130*xl* genetic analyser using a 16 capillary 36 cm array with either POP-4 or POP-7 separation matrix (all Life Technologies, Paisley, UK). Genetic analysers were calibrated for the G5 dyeset. GeneScan 1200 LIZ standard was used as internal sizing reference (Life Technologies, Paisley, UK). Fragment analysis samples contained 1 μL amplified DNA, 0.5 μL 1200 LIZ standard and 8.5 μL Hi-Di formamide (Life Technologies, Paisley, UK). Samples were injected at 5 kV for 5 sec and resolved using a separation voltage of 6.5 kV for 103 min. Major peaks in fluorescent signal were imported into BioNumerics v.5.1 software (Applied Maths, Sint-Martens-Latem) to complete the method validation. Fragments were initially sized using either PeakScanner v.1.0 or GeneMapper v.4.0 software (both Life Technologies, Paisley, UK), or were imported into BioNumerics directly. All signals with a height <10% that of the highest peak in the individual profile were excluded (as these were considered background rather than evidence of a major DNA fragment). For peaks <1.5bp different in size, the lower intensity peak was also excluded [18].

### Consensus method validation

**Fig 1:**
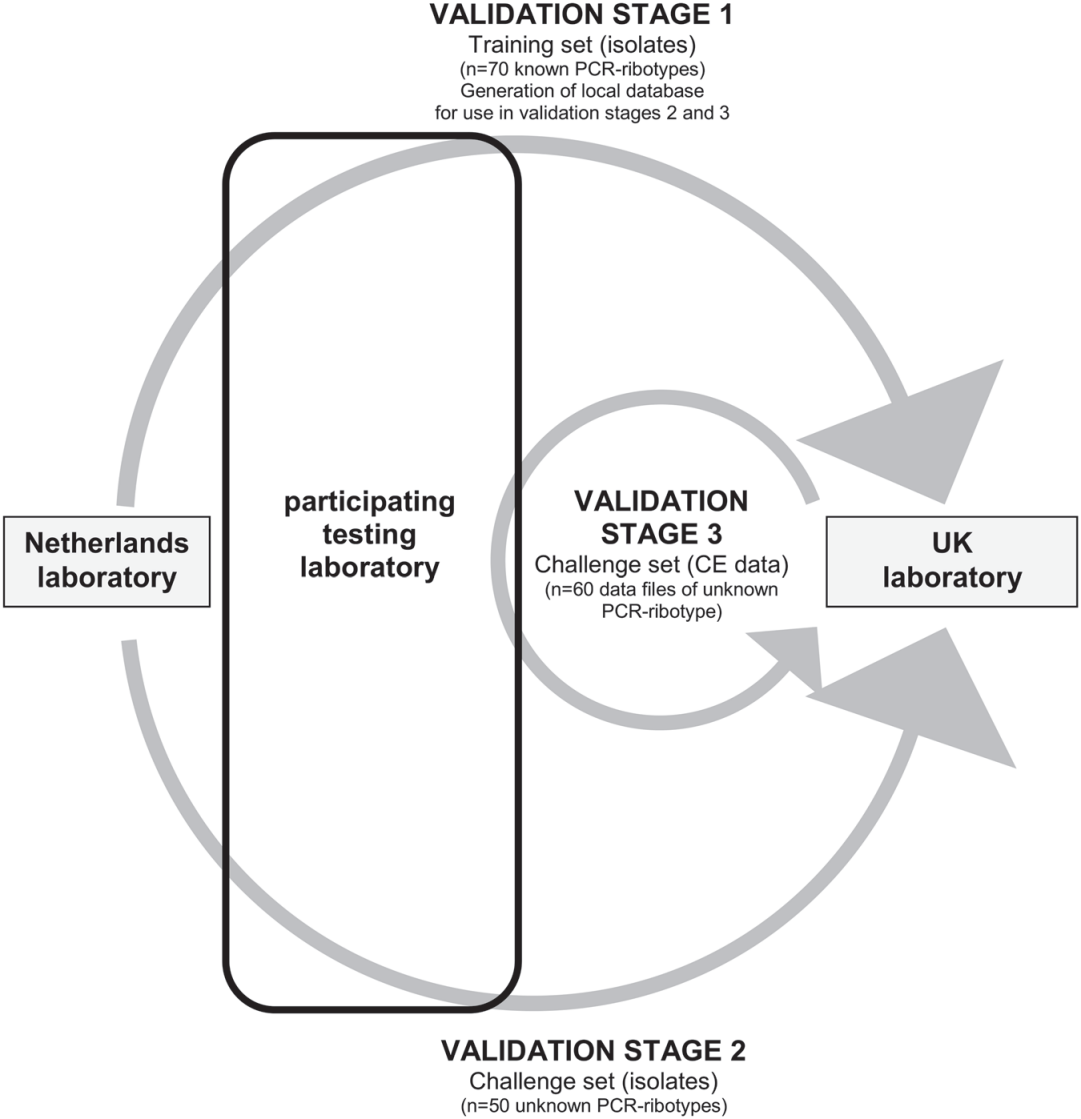
Process for multi-centre consensus method validation. **STAGE 1:** 70 well characterised ribotypes disseminated from Netherlands to each laboratory for ribotyping (data (i) held locally for future comparsion/ribotype assignment, (ii) sent to UK laboratory); **STAGE 2:** 50 anonymised isolates disseminated from Netherlands to each laboratory for ribotype identification (assignments sent to UK laboratory for analysis); **STAGE 3:** 60 anonymised data files disseminated from UK to each laboratory for ribotype identification (assignments sent to UK for analysis).

Multi-centre validation of the proposed consensus method is outlined in Fig. 1. Briefly, validation was performed in three stages:

**VALIDATION STAGE 1: To examine inter-laboratory variation and reproducibility, and establish local reference databases.** The panel of 70 known distinct PCR-ribotypes was distributed centrally (from the Netherlands laboratory) to all other participating national surveillance laboratories (training set). CE-ribotyping was performed at each centre, using the proposed consensus method, and data were held locally in BioNumerics. These training data were used for all subsequent PCR-ribotype identifications throughout the validation process, and also returned to the UK laboratory for analysis and use in validation stage 3.

**VALIDATION STAGE 2: To examine accuracy of PCR-ribotype identification from locally generated data.** A panel of 50 isolates were anonymised and distributed centrally from the Netherlands laboratory (isolate challenge set). CE-ribotyping was performed at each centre using the proposed consensus method. A PCR-ribotype was assigned to each isolate after DNA profile comparison with the data generated using the training set of isolates in validation stage 1 (BioNumerics). PCR-ribotype assignments from each centre were returned to the UK laboratory for analysis. PCR-ribotype identities for the 50 isolates were then circulated by the Netherlands laboratory.

**VALIDATION STAGE 3: To examine portability of data and accuracy of PCR-ribotype identification from centrally generated electronic data.** A dataset of sized fragments for 60 PCR-ribotypes was extracted from data returned to the UK laboratory in validation stage 1 (15 from each participating centre). Data files were anonymised and distributed by email to all other participating laboratories (data challenge set). A PCR-ribotype was assigned to each data file after DNA profile comparison with the data generated using the training set of isolates in validation stage 1 (BioNumerics). PCR-ribotype assignments from each centre were returned to the UK laboratory for analysis.

## Results

### VALIDATION STAGE ONE—training set

**Fig 2:**
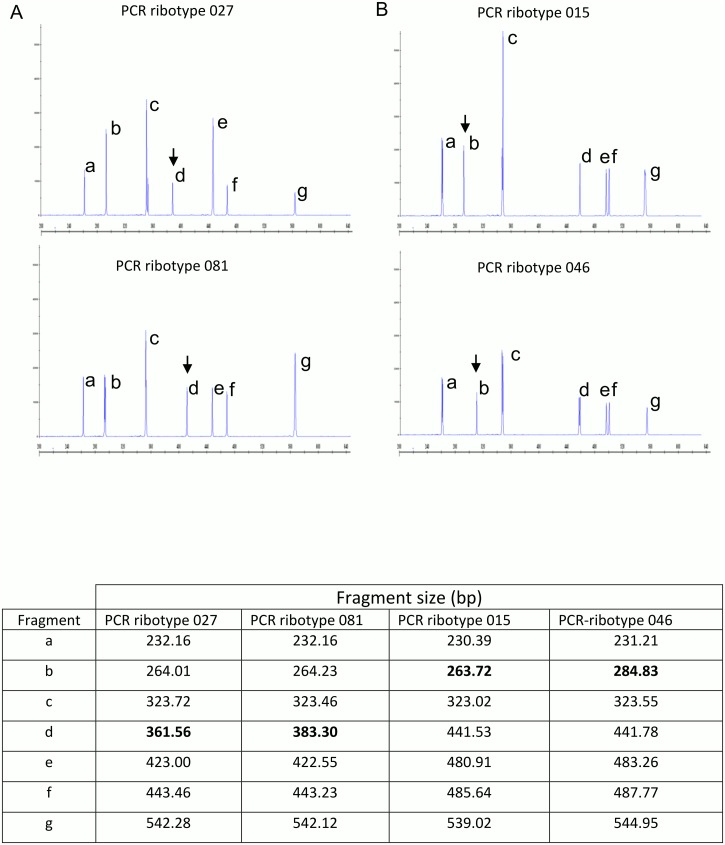
PCR-ribotypes with very similar profiles: (a) ribotypes 027 and 081 and (b) ribotypes 015 and 046. PCR-ribotypes 027 and 081 differ from one another by only a ~20bp difference at position d. Similarly PCR-ribotypes 015 and 046 differ by only a ~20bp difference at position b. Discriminating features between these very similar profiles are indicated (arrows) and associated fragment sizes are highlighted in bold. Relative fragment size was the only parameter used to discriminate between ribotype profiles. Relative peak heights (relative fluorescent units, y-axis) within profiles lacked reproducibility for some ribotypes and therefore this parameter was not used.

The consensus CE-ribotyping method was applied to the panel of 70 well characterised ribotypes at all four participating centres. Each laboratory generated between 5 and 11 fragments per ribotype, ranging in size from 223–667 bp. A high level of profile similarity was observed between several ribotypes in the panel (Fig. 2). A high level of reproducibility was observed; the standard deviations (SDs) for fragment sizes reported from all four laboratories on each individual fragment ranged from 0.14–3.8 bp (mean 1.25, median 1.17). The maximum reported difference in individual fragment size ranged from 0.3–8.4 bp (mean 2.6 bp, median 2.5 bp) (Table 3). The magnitude of inter-laboratory variation was clearly associated with increasing fragment size. The maximum reported size differences of the smallest and largest fragments per ribotype profile were significantly different (Mann Whitney U test, (*p<0*.*05*); median SD of smallest and largest fragments per ribotype profile: 0.39 and 2.13, respectively). No significant differences were observed in the fragment sizes generated on the 3100 versus the 3130*xl* automated sequencing instruments.

**Table 3:** PCR-ribotypes associated with minimum and maximum differences in DNA fragment sizes reported across participating centres.

Fragment^a^	PCR ribotype 084				PCR ribotype 064			
	Fragment size range^b^		Sizediff^c^	SD^d^	Fragment size range^b^		Sizediff^c^	SD^d^
	min	max			min	max		
a	231.92	232.25	**0.33**	**0.14**	231.70	232.52	0.82	0.35
b	283.70	285.35	1.65	0.71	262.60	264.02	1.42	0.59
c	325.59	328.25	2.66	1.23	283.56	285.25	1.69	0.72
d	364.54	368.02	3.48	1.60	322.27	326.23	3.96	1.63
e	421.86	424.45	2.59	1.06	482.02	485.29	3.27	1.45
f	479.81	483.98	4.17	1.95	540.56	545.33	4.77	2.36
g	482.12	485.53	3.41	1.51	542.60	547.01	4.41	2.04
h					544.91	549.53	4.62	2.18
i					658.55	666.92	**8.37**	**3.79**

minimum and maximum values are in displayed in bold

^a^designated fragment in ribotype profile

^b^minimum and maximum sizes reported for a specific fragment

^c^difference between maximum and minimum reported size for a specific fragment

^d^standard deviation between reported sizes for a specific fragment

### VALIDATION STAGE TWO—isolate challenge set

The CE-ribotyping method was applied to the panel of 50 anonymised isolates distributed from the Netherlands laboratory (isolate challenge set). Formal PCR-ribotype identification was performed by each laboratory using training data generated in validation stage one. A list of PCR-ribotypes represented in this challenge set and PCR-ribotyping performance scores for each centre are shown in Tables 1 and 4.

**Table 4:** Laboratory performance for PCR-ribotype assignment in validation stages two and three.

	Number of correctly assigned PCR-ribotypes				
	Centre A	Centre B	Centre C	Centre D	All Centres
**Validation stage 2**	50/50(100.0%)	49/50(98.0%)	47/50(94.0%)	49/50(98.0%)	195/200(97.5%)
**Validation stage 3**	60/60(100.0%)	57/60(95.0%)	60/60(100.0%)	60/60(100.0%)	237/240(98.8%)
		60/60(100%)*			240/240(100%)*
**All tests**	110/110(100.0%)	106/110(96.4%)	107/110(97.3%)	109/110(99.1%)	432/440(98.2%)
		109/110(99.1%)*			435/440(98.8%)*

*Analysis of discrepancies highlighted an error during database identification at centre B (as opposed to inconsistencies with the prescribed method, or data quality). A second identification attempt using the same profile data increased the number of correctly identified PCR-ribotype profiles in both validation stage 3 and the overall study to 240/240 (100%) and 435/440 (98.9%) respectively.

Laboratories identified the correct PCR-ribotype in 195/200 (97.5%) of cases (or correctly stated that the profile generated was not recognisable, based on the training data generated in validation stage one). Forty-five of 50 (90.0%) PCR-ribotypes were discriminated successfully by all four centres; all PCR-ribotypes were discriminated successfully by a minimum of three centres. Analysis of the discrepancies revealed that identification failures (n = 5) were not associated with any particular testing centre or any specific PCR-ribotype (Table 5). Data consistent with PCR-ribotype 064 were generated in place of PCR-ribotype 029 by centre B indicating likely transcription of the test isolate. The profile for PCR-ribotype 035 was obscured with additional peaks consistent with sample contamination at centre C. PCR-ribotypes 058 and 118 were associated with amplification failure at centre C and likely to represent attempted testing of an organism other than *C*. *difficile*. An error in the construction of the challenge set resulted in the circulation of PCR-ribotype 036 in place of PCR-ribotype 198. Centre D successfully identified the isolate as PCR-ribotype 036, but should have technically returned a result of “unidentified” as PCR-ribotype 036 was not present in the training set. All test failures highlighted errors associated with the manipulation of test isolates as opposed to inconsistencies with the prescribed method, or data quality.

**Table 5:** Analysis of five discrepant results generated in validation stage two (isolate challenge set).

	PCR-ribotype	Testing Centre	Reported result	Comments
**1**	029	B	064	probable isolate transcription
**2**	035	C	unidentified	probable contamination of sample
**3**	058	C	no profile generated	test isolate unlikely to be *C*. *difficile*
**4**	118	C	no profile generated	test isolate unlikely to be *C*. *difficile*
**5**	198	D	036	isolate of incorrect PCR-ribotype circulated in challenge set

Only 5 identification failures were present in validation stage 2, and were not associated with any particular testing centre or any specific PCR-ribotype. All failures highlighted errors associated with the manipulation of test isolates as opposed to inconsistencies with the prescribed method, or data quality.

### VALIDATION STAGE THREE—data challenge set

A dataset of sized fragments for 60 blinded ribotypes was distributed electronically from the UK laboratory. Formal PCR-ribotype identification was performed by each laboratory using training data generated in validation stage one. A list of PCR-ribotypes represented in this challenge set and PCR-ribotyping performance scores for each centre are shown in Tables 1 and 4. Laboratories identified the correct ribotype in 237/240 (98.8%) cases. All four centres correctly identified 57/60 (95%) ribotype profiles. All isolates were correctly identified by a minimum of three centres. Failure to identify the correct ribotype was only associated with Centre B for the remaining three isolates. Analysis of the discrepancies revealed that an incomplete set of PCR-ribotype profiles (generated from the training set) had been used to formally identify PCR-ribotypes at this centre, resulting in three unidentified profiles. Using the same profile data, a second identification attempt (containing a full set of PCR-ribotype reference profiles) was performed at centre B and 60/60 (100%) PCR-ribotype profiles were correctly identified. This process highlighted an error during database identification at a single centre as opposed to inconsistencies with the prescribed method, or data quality. Following analysis of the discrepancies, the number of correctly identified PCR-ribotype profiles in both validation stage 3 and the overall study was increased to 240/240 (100%) and 435/440 (98.9%), respectively.

## Discussion

Increased awareness of CDI is accompanied by the need to develop a consensus typing and analysis method that will allow the establishment of a standardised nomenclature and greater understanding of *C*. *difficile* epidemiology. This should be robust, reliable and generate high resolution, portable data, and function at local, national and international levels. Existing reports involving CE-ribotyping have represented only single-centre studies and inter-laboratory variability has not been formally assessed [18, 20]. We report a high level of reproducibility and accuracy associated with a consensus CE-ribotyping protocol. A maximum SD of only ±3.8 bp was recorded in individual fragment sizes, and 98.2% blinded ribotypes were correctly identified at four international ribotyping centres spanning Europe and North America (rising to 98.8% after analysing the discrepancies). Our results demonstrate that this consensus protocol generates sufficiently low level inter-laboratory variation to support accurate PCR-ribotyping for international *C*. *difficile* surveillance.

Indra *et al*. first combined conventional PCR-ribotyping and capillary based electrophoresis techniques (CE-ribotyping) and demonstrated superiority of this approach over the conventional agarose gel-based method [18]. More recently, Xiao *et al*. reported that the performance of capillary-electrophoresis exceeded that of a commercial high performance electrophoresis system when applied to PCR-ribotyping. [20]. Several epidemiological studies have been conducted using subtly different CE-ribotyping protocols, making future inter-laboratory comparison of results difficult [18, 20, 24, 25]. We hope that the provision of a consensus CE-ribotyping protocol will encourage laboratories to use the technique in the knowledge that data will be directly comparable across centres.

We have shown that a basic crude DNA extraction method is sufficient to demonstrate good reproducibility of the system. However, many laboratories have more sophisticated higher-throughput DNA extraction platforms, which invariably yield good quality DNA, and these could be expected to be viable alternatives. Several different sets of PCR primers have been reported for use in PCR-ribotyping in recent years [15, 17, 26]. The primers originally described by Stubbs *et al*. unfortunately had a 4 bp mismatch in the 23S reverse primer [15] and were subsequently redesigned [17]. It is generally accepted that these primer sets offer equivalent levels of performance and discrimination, but generate different relative fragment lengths. Bidet *et al*. primers generate fragments ~34 bp shorter than those designed by Stubbs *et al*., and ~53 bp shorter than those designed by Sadeghifard *et al*. The loss of accuracy associated with sizing larger DNA fragments when using electrophoresis should ideally be minimised, and the selection of primers that generate smaller fragments would therefore be preferable. In order to avoid potential primer:template mismatches and minimise inter-laboratory variation, the primers designed by Bidet *et al*., were selected for use in this consensus method [17]. It is important that standardized protocols for multi-centre surveillance can be easily adopted by participating laboratories. PCR consumables can be acquired or changed relatively easily, but access to specific DNA sequencing instrumentation can present difficulties. It is therefore essential that a standardised protocol can operate on more than one instrument type without significant variation in ribotype profiles. Our study has incorporated the use of two different sequencing instruments with little effect on PCR-ribotype recognition or fragment-size variation. These results suggest that laboratories can adopt the consensus protocol for use on different automated sequencers; further validation is required to confirm this issue.

Previous reports using CE-ribotyping have called for validation of the technique using internationally accepted reference strains [18, 20]. The standardised consensus method used here was tested on a well characterised collection of 70 different PCR-ribotypes [22, 23] to evaluate the performance of the protocol against prevalent and clinically relevant PCR-ribotypes. Data on this set of strains are available on-line in a NCBI BioProject database to facilitate working in this field (http://www.ncbi.nlm.nih.gov/bioproject/248340). Several examples of profiles that shared a very high level of similarity were incorporated in this study (eg. 027 and 081; 015 and 046; 106 and 174; 014 and 020) (Fig. 2). CE ribotyping was able to consistently discriminate between all these isolates. It is generally accepted that such discrimination is difficult with conventional agarose gel-based PCR-ribotyping unless gel quality is particularly high. The number of unique PCR-ribotypes has expanded rapidly in recent years and now exceeds 650 (Fawley, Wilcox, unpublished). As this library expands, the number of ribotypes with similar profile is likely to increase. In turn, the interpretation of PCR-ribotype profiles will become more complex. CE-ribotyping offers a solution for future ribotyping strategies by offering accurate fragment sizing and increased discrimination between ribotypes, including those with similar profiles that can be confused using conventional agarose gel-based PCR-ribotyping. CE-ribotyping has already confirmed reproducible differences in DNA profile of isolates previously assigned to the same ribotype using agarose gel electrophoresis, most notably those associated with ribotypes 014, 001 and 027 [18, 20, 27]. Multi-locus sequence typing studies have further shown that individual ribotypes tend to be associated with a single ST-type, but that a small number are associated with multiple ST-types [22, 27, 28].

CE-ribotyping represents a cost-effective technique to further discriminate between ribotypes of very similar DNA profile in order to further understand phylogenetic relationships. The consumables price per test for PCR-ribotyping by CE-method and by conventional agarose gel-based method are comparable (under EUR 10) [19]. Current opinion would indicate that the largest barrier to widespread adoption of CE-ribotyping remains the acquisition of an automated sequencing instrument. However, such instruments are now in widespread use; most medical and academic institutions use automated sequencing technology and many offer access to their instruments (on or off site) on a service provision basis. In this way CE-ribotyping could reasonably be performed in any laboratory and need not be reserved for larger reference centres.

Our results suggest that data generated using the consensus method are fully portable; 98.8% (rising to 100% after analysing the discrepancies) of ribotype profiles were correctly identified when data were exchanged electronically between participating laboratories. Hitherto, surveillance programs have been impeded due to the inability of laboratories to exchange typing data efficiently. Notably, understanding the spread of NAP1/BI/PCR ribotype 027 strains across N. America and Europe was affected by inefficient inter-laboratory exchange of PCR-ribotyping data, involving the laborious exchange of bacterial isolates to establish significant epidemiological links [2, 6]. We have demonstrated that a standardised CE-ribotyping protocol allows easy electronic exchange of typing data, which could streamline communication and so reduce the time for countries/regions to become aware of new and emerging ribotypes.

There have been calls for improvements to PCR-ribotype nomenclature [19, 29]. Stubbs *et al*. first assembled a library of PCR-ribotypes [16] but, while this collection has expanded to >650 types, lack of direct access to this library has led some to use local ribotype nomenclatures. These have only local immediate applicability. The UK library was developed using agarose gel-based ribotyping, and further development is hindered by interpretation issues associated with this technique. Standardised and fully portable ribotyping data would be a significant step forwards to consolidating a single library of *C*. *difficile* reference strains, which would bring clarity for researchers and those involved in surveillance of this important pathogen.
